# Bioactive Compounds of Green Phenolic Extracts Obtained via Microwave-Assisted Extraction of *Sideritis* Species Grown in Greece

**DOI:** 10.3390/molecules29235612

**Published:** 2024-11-27

**Authors:** Elisavet Bouloumpasi, Anna Koskeridou, Maria Irakli, Anastasia Karioti, Nektaria Tsivelika, Paschalina Chatzopoulou

**Affiliations:** 1 Hellenic Agricultural Organization—Dimitra, Institute of Plant Breeding and Genetic Resources, 57001 Thessaloniki, Greece; ebouloum@vo.duth.gr (E.B.); riatsivel@gmail.com (N.T.); 2 Laboratory of Pharmacognosy, School of Pharmacy, Aristotle University of Thessaloniki, University Campus, 54124 Thessaloniki, Greece; annatktr@hotmail.com (A.K.); akarioti@pharm.auth.gr (A.K.)

**Keywords:** mountain tea, microwave-assisted extraction, phenolic profile, antioxidant activity, *Sideritis* species, isoscutellarein, hypolaetin

## Abstract

The purpose of the present study was to compare the polyphenolic compounds extracted from five *Sideritis* species grown in Greece; *S. scardica*, *S. clandestina*, *S. raeseri*, *S. euboea*, and *S. syriaca,* using the Microwave-Assisted Extraction (MAE) process. To maximize the extraction yield (EY), total phenolic compounds (TPC), hypolaetin (HYP) and isoscutellarein (ISC), derivative contents (target phenolics), the response surface methodology was used for *S. scardica*. A Box–Behnken design was undertaken to study the effect of ethanol concentration (30–100%), extraction temperature (40–100 °C), and extraction time (5–25 min) on the responses. The optimal MAE parameters were 87.9% (*v*/*v*) ethanol, 25 min, and 100 °C. Under these conditions, there was a good agreement between experimental and predicted values, indicating the reliability of the predictions for *Sideritis* extracts. Phenolic compounds were then extracted under these conditions, from the five *Sideritis* species under investigation. The TPC, total flavonoid content (TFC), antioxidant activity based on DPPH, ABTS, and FRAP assays as well as the phenolic profile of different *Sideritis* extracts, evaluated via HPLC-DAD-MS, were compared. A similar phenolic profile was observed among the five *Sideritis* species, with HYP and ISC derivatives showing variations in their contents as a function of *Sideritis* species. MAE *Sideritis* extracts could be considered green and natural antioxidants for medicinal, cosmetic, and food purposes, accompanied by sustainable approaches.

## 1. Introduction

The genus *Sideritis* (S) is composed of more than 150 species, with numerous taxa widely distributed in southeast European and Mediterranean countries. The following *Sideritis* species grown in Greece are classified to the sect. Empedoclia (RAFIN) Bentham are perennial, with *S. raeseri* and *S. scardica* being the most abundant and distributed on continental part of the country, whilst the species *S. syriaca* L. subsp. *syriaca*, *S. raeseri* subsp. *attica* (HELDR.), *S. clandestina* (BOISS. & HELDR.), *S. euboea* HELDR., and *S. perfoliata* subsp. *athoa* (Papanic. & Kokkini), are endemic [1,2,3,4]. The plant was known in ancient Greece, and it is mentioned by Theophrastus and Dioscorides. The name Sideritis (ironwort) originates from the Greek word for iron (σιδηρος), since it was believed in antiquity that it was effective at wound healing from trauma caused by iron weapons. *Sideritis syriaca* growing in Creta, is historically known with the common name “malotira”, a synthetic word from the Italians; *male* means ailment/illness, and *tirare* means to pull, concluding the action of withdrawing the symptoms of illness [5]. Gonzales Burgos et al. [6], Todorova et al. [7], Avena et al. [8] Bojovic et al. [9], Yaneva et al., [10] and Romanucci et al. [11] in their reviews, mention common names, traditional uses of various *Sideritis* species in the folk medicine, and methods for preparation of herbal remedies in several countries.

Plants of *Sideritis* species are traditionally used as herbal tea, with the common name “Mountain tea”, which has a pleasant aroma, and is known for its health benefits in folk medicine for treating asthma and coughs, bronchitis, common cold, preventing anemia, and being analgesic, sedative, diuretic, diaphoretic, antimicrobial, antibacterial, antioxidant, and anti-inflammatory etc. [6,7,12]. Consequently, many historical data supported the traditional uses of several *Sideritis* taxa [5]. The European Medicines Agency (EMA) has approved the traditional uses of herba *S. scardica* Griseb., *S. clandestina* (Bory & Chaub.) Hayek, *S. raeseri* Boiss. & Heldr., and *S. syriaca* L., for the relief of cough associated with cold, and of mild gastrointestinal discomfort [13]. Recent pharmacological studies have reported antimicrobial [14], anti-inflammatory [15], antineuropathic [15], antioxidant [16], antidiabetic [17], anxiolytic [18], and gastroprotective properties [19], etc., while during the last decades much research focused on the effectiveness of various *Sideritis* extracts on neurological disorders and neurodegenerative diseases [12,20,21,22].

Pharmacological properties are attributed to the phytochemical profile of plant extracts. In this context numerous works have demonstrated that diterpenes, polyphenols, i.e., flavonoids, phenolic acids, and essential oils, are the primary bioactive substances, found in different *Sideritis* species [16,23,24,25,26,27]. Derivatives of hypolaetin (HYP) and isoscutellarein (ISC) were identified as the main flavonoids in central and eastern Mediterranean *Sideritis* species [24,25,26,27,28]. More specifically, Petreska et al. [24] and Axiotis et al. [25] have reported phenylethanoid glycosides and flavonoid acetylglycosides in methanol extracts from various *Sideritis* species. Also, Gabrieli et al. [26] recovered nine 7-O-allosyl glucosides of 5,8-dihydroxy substituted flavones from the methanol extract of the aerial parts of *S. raeseri* subsp. *raeseri*. Armata et al. [27] identified esters of quinic acid, chlorogenic acid, apigenin and several isoscutellarein (ISC) derivatives in extracts of *S. syriaca* ssp. *syriaca*, and Irakli et al. [16] found nine phenolic acids (mainly hydroxycinanamic acids) i.e., protocatechuic acid, 4-hydroxybenzoic acid, chlorogenic acid, vanillic acid, caffeic acid, syringic acid, p-coumaric acid, ferulic acid, and cinnamic acid in *S. scardica* infusion.

The extraction process is essential for obtaining bioactive compounds from plants. Traditional methods including decoction, maceration, infusion, digestion, percolation, and Soxhlet extraction to ensure the quality of the extracts with low degradation products, however, traditional protocols cause environmental impact along with time-consuming, and high-energy consumption processes. Therefore, eco-friendly and advanced methods are preferable to enhance the safety of final products and improve the extraction process. This can be achieved by using various techniques and among them Ultrasound-Assisted Extraction (UAE), Accelerated Solvent Extraction (ASE), and Microwave-Assisted Extraction (MAE), as alternatives to traditional methods, for the extraction of bioactive compounds from plant-based matrices and products, in response to the potential use of natural ingredients in different products, considering environmental issues as well [28]. These methods respect the principles of green chemistry that focus on reducing or eliminating hazardous substances, speeding up the extraction process, minimizing their negative impact on human health and the environment, and maintain at the same time, high-quality analytical results. Therefore, the selection of highly sustainable and environmentally friendly extraction procedures is a crucial step in natural products.

One of the most interesting emerging techniques of extraction is MAE that exploits the interaction between microwave radiation, which generates electromagnetic waves with a frequency typically ranging from 300 MHz to 300 GHz. The microwave energy adsorbed by the material causes the displacement of polar molecules creating dipole rotation, driving the molecules to align to the existing electric field, leading to internal heat generation [29]. The direct interaction between a sample and electromagnetic radiation improves the extraction efficiency and enhances analyte recovery, leading to rapid and increased solubility of target compounds such as phenolic compounds. Therefore, MAE can be considered a green analytical method that contributes to a more eco-friendly and sustainable approach to environmental and energy management [30]. Recently, MAE has gained popularity due to its benefits of improved extraction efficiency, shorter extraction time, low solvent consumption, higher extraction rate, saving of electrical energy, low costs of the equipment, and high potential for automation [28].

In analytical chemistry applications, it has been observed that MAE achieved similar or greater yields than UAE in the case of phenolic compounds from plant matrices [31,32]. With regard to *Sideritis*, previous studies [33] using older MAE systems reported similar or lower yield for MAE extraction than UAE, depending on the conditions of the extraction. During UAE, cavitation promotes mass transfer kinetics and equilibrium and destabilizes the cell wall, making it ultrasonication advantageous [29]. However, UAE may cause thermal degradation of the heat-labile compound and low reproducibility due to the aging of the device that may decrease ultrasound intensity [28]. Recently, the extraction of polyphenols from a variety of aromatic plants has been accomplished under MAE, i.e., oregano [34,35], rosemary [31,36], *Sideritis* species [37,38]; *Hyssopus officinalis* [39]; *Coriandrum sativum* and *Crocus sativus* [40]; *Origanum dictamnus* and *Origanum majorana* [41,42], lavender [43], and basil [44], etc. While few studies have focused on the optimization methodology to obtain phenolic-rich extracts from *Sideritis* species [45,46,47], no work has emphasized the optimization of polyphenols extracted under MAE from *Sideritis* species, based specifically on the contents of HYP and ISC derivatives, which are the characteristic compounds in *Sideritis*. Sarakatsianos et al. [37] studied and optimized MAE extraction conditions of phenolic constituents from *S. raeseri* and *S. scardica* recording total polyphenols or flavonoid content as final responses.

Due to the multiple biological activities and the contemporary or potential uses of *Sideritis* extracts in various products such as functional foods, cosmetics, bioactive ingredients, herbal drugs, etc., the purpose of the present study was to compare the polyphenolic compounds extracted from five Greek *Sideritis* species; *S. scardica*, *S. clandestina* subsp. *peloponnesiaca*, *S. raeseri*, *S. euboea*, and *S. syriaca*, using the MAE process. The effects of various ethanol concentration, time, and temperature extraction were evaluated in *S. scardica* via the response surface methodology (RSM), to determine the optimal MAE extraction parameters. Under optimal conditions, the concentration of the primary phenolics, such as ISC and HYP derivatives, in the produced extracts, was used for assessing the extraction technique’s efficiency and subsequently was applied for the evaluation of their concentration in the studied *Sideritis* species.

## 2. Results and Discussion

### 2.1. Preliminary Studies

The genus *Sideritis* (Lamiaceae family) comprises more than 150 species worldwide with varying bioactive compositions [46]. Due to the complicated phytochemical profile of *Sideritis* plants, and in order to achieve high-yield recovery of highly bioactive compounds, it is necessary to optimize the MAE extraction conditions. *S. scardica* was selected as the substrate for experimental trials as it is a common species in other countries including Bulgaria, North Macedonia, and Turkey; among the four taxa included in the European Union herbal monograph for *Sideritis* herba, it is the most extensively studied. As for all the extraction techniques, the optimal conditions depend highly on the matrix characteristics and the targeted analytes to extract. The application of RSM is considered a faster and more structured approach to designing experiments for the investigation of independent factors and response variables as well as various interactions that may exist between the independent variables [32]. The MAE process has several advantages for optimizing specific responses influenced by variables such as extraction solvent, extraction time, and temperature [47].

A preliminary study was applied to select the range of the independent factors, taking into account earlier studies [33,36,38]. Solvent type and concentration substantially affect the extraction efficiency [31,48]. Although methanol has been reported to yield a greater extraction efficiency for TPC, it is not preferred when the extracts are going to be used for nutraceutical or food grade purposes, due to its toxicity issues [48]. In this study, ethanol was selected as the extraction solvent, as it is categorized as GRAS. Ethanol concentration also plays a critical role in the extraction of bioactive compounds from natural products. Water was chosen as a co-solvent as it is reported that it is more detrimental to the extraction selectivity of polar compounds. All the extraction experiments were conducted at the same solvent-to-solid ratio of 80:1 (mL:g), since Alipieva et al. [33] showed that increasing the plant material/solvent ratio from 1:15 to 1: 50 *w*/*v* (g/mL) had a positive influence on the MAE extraction yield from *Sideritis*, while Sarakatsianos et al. [37] used a ratio 1:60 g/mL in their study and Akbaba et al. used 1:80 g/mL. With regard to the extraction time, previous research results reported a microwave radiation duration of 10 min, followed by 10 min cooling [37], or a total duration of 18 to 30 min [38], while temperature ranged between 60 and 85 °C [38].

In our study, we applied a Box–Behnken experimental design (BBD) with three variables tested at three levels (Table 1) in order to obtain an extract of *S. scardica* with the highest extraction yield and the richest phenolic content, as well as the highest content of the individual bioactive compounds (ISC and HYP derivatives).

**Figure 1:**
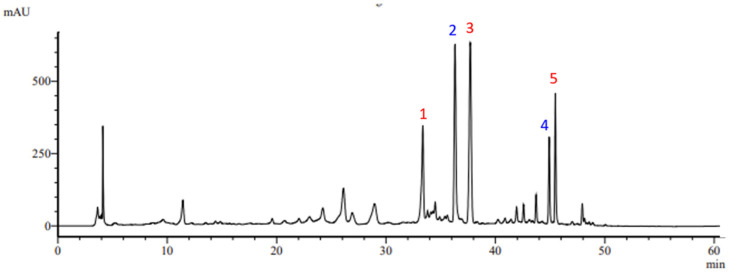
A representative chromatogram at 280 nm of S. scardica extract obtained using MAE. Peaks 1, 3, and 5 (red color) correspond to hypolaetin derivatives and peaks 2 and 4 (blue color) to isoscutellarein derivatives.

A representative chromatograph at 280 nm of phenolic extract of *S. scardica* obtained via the MAE is illustrated in Figure 1. The main phenolic compounds in *S. scardica* extracts were tentatively identified and characterized based on their retention times, UV spectra, and MS spectra ([M−H]^−^ ions), as confirmed by literature reports [24,25,33,45,46,49,50,51,52,53]. A total of five peaks were detected as the major phenolic compounds in all *S. scardica* extracts. Peak 1 (hypolaetin-7-O-[6‴-O-acetyl]-allosyl(1→2)glucoside) with [M−H]^−^ at *m*/*z* 667, peak 3 (4′-O-methylhypolaetin 7-O-[6‴-O-acetyl]-allosyl(1→2)-glucoside) with [M−H]^−^ at *m*/*z* 681, and peak 5 (3-O-methylhypolaetin 7-O-[6‴-O-acetyl]-allosyl-(1→2)-[6″-O-acetyl]-glucoside) with [M−H]^−^ at *m*/*z* 723 corresponded to hypolaetin derivatives (HYP DER). Peak 2 (isoscutellarein-7-O-allosyl-(1→2)-[6″-O-acetyl]-glucoside) with [M−H]^−^ at *m*/*z* 651 and peak 4 (isoscutellarein-7-O-[6‴-O-acetyl]-allosyl-(1→2)-[6″-O-acetyl]-glucoside) with [M−H]^−^ at *m*/*z* 693 have been characterized as isoscutellarein derivatives (ISC DER) (Figure 1). The contents of HYP DER (sum of peaks 1, 3, and 5) and ISC DER (sum of peaks 2 and 4) were recorded as one of the responses along with the extraction yield (EY) and total phenolic content (TPC).

### 2.2. Modeling of the MAE Process for the Extraction of Phenolics from S. scardica

The experimental data of *S. scardica* extracts in connection with the independent factors are presented in Table 2. The extraction yield ranged from 5.6 to 21.6%, while the TPC values ranged from 90.00 to 125.01 mg GAE/g dw. ISC and HYP derivatives ranged from 54.02 to 92.03 mg/g and 72.03 to 135.61 mg/g, respectively. Table 3 shows the ANOVA results for the effects of ethanol concentration, time extraction, and temperature extraction via MAE on EY, TPC, and ISC DER and HYP DER of *S. scardica*.

Linear and quadratic terms of ethanol concentration, as well as quadratic terms of time extraction had a statistically significant influence on all investigated responses, while interaction between ethanol concentration and time extraction did not have statistically significant influence. It was clearly demonstrated that solvent polarity is a parameter that has a pronounced effect on phenolic compound extraction from the *S. scardica* studied. Linear term of extraction time seems to have a significant effect only on TPC, while linear term of temperature extraction had a significant effect on TPC and HYP DER contents of *S. scardica* extracts. In the interaction effects, ethanol concentration/temperature extraction (*X*_1_*X*_3_) had a significant effect on TPC (*p* ≤ 0.05), ISC DER (*p* < 0.05) and HYP DER (*p* < 0.01), while time extraction/temperature extraction (*X*_2_*X*_3_) had a significant effect on TPC.

Taking into account only the significant terms, the following regression equations were generated for EY (Equation (1)), TPC (Equation (2)), ISC DER (Equation (3)), and HYP DER (Equation (4)), based on the relationship of ethanol concentration, (*X*_1_), time extraction (*X*_2_), and temperature extraction (*X*_3_) in terms of their coded units:

*EY* = 20.24 − 4.06*X*_1_ + 0.4779*X*_2_ + 0.9645*X*_3_ − 6.05*X*_1_^2^ − 2.95*X*_2_^2^ − 1.82*X*_3_^2^ (1)

*TPC* = 91.83 + 5.95*X*_1_ + 3.26*X*_2_ + 5.13*X*_3_ + 12.32*X*_1_^2^ + 6.78*X*_2_^2^ + 3.45*X*_1_*X*_2_ + 6.78*X*_1_*X*_3_ + 6.36*X*_2_*X*_3_ (2)

*ISC DER* = 61.14 + 10.20*X*_1_ + 1.75*X*_2_ + 0.0563*X*_3_ + 7.04*X*_1_^2^ + 8.08*X*_2_^2^ 4.23*X*_3_^2^ + 7.59*X*_1_*X*_3_ (3)

*HYP DER* = 92.19 + 11.98*X*_1_ + 8.72*X*_3_ + 13.83*X*_1_*X*_3_ (4)

The models corresponding to the EY, TPC, ISC DER, and HYP DER responses, indicated a significant (*p* < 0.01) relationship between the responses and the independent variables. Similarly, the adequacy of the model was evaluated via the coefficient of determination (R^2^), and via *p*-values for the lack of fit testing. The *p* values of all lack of fit were higher than 0.05, implying that all quadratic polynomial models were successfully fitted the design and were reliable and proper for predicting the relevant responses [50]. The particularly high value of R^2^ for EY (0.9361), as well as high values of the same parameter for TPC (0.9768), ISC DER (0.9730), and HYP DER (0.9654), indicated that only a very small percentage (0.06%) of the total variation remains unexplained by these models. In addition, the adjusted coefficients of determination (R^2^_adj_) for the EY, TPC, ISC DER, and HYP DER models were 0.9361, 0.9351, 0.9245, and 0.9031, respectively, indicating close agreement between the experimental and predicted values. These findings suggest that the current model is sufficient to fit the experimental data.

### 2.3. Analysis of Plots Describing MAE Factors Effect

**Figure 2:**
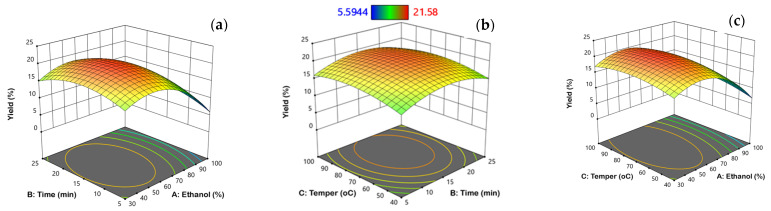

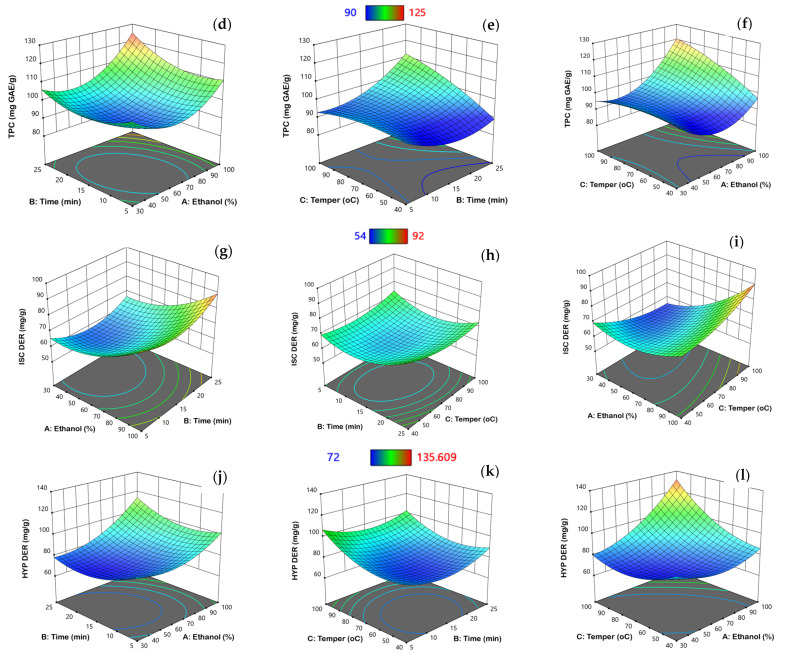
Response surface plots to illustrate interactive effects on yield (**a**–**c**), total phenolic content, TPC (**d**–**f**), isoscutellarein derivatives, ISC DER (**g**–**i**), and hypolaetin derivatives. HYP DER (**j**–**l**) was a function of two independent variables, keeping the other at the central point.

The effects of operational factors on MAE of extraction yield, TPC, and contents of ISC DER and HYP DER of *S. scardica* extracts were investigated. Figure 2 shows the effects of operational parameters on yield, TPC, ISC, and HYP derivative contents, in the form of three-dimensional (3D) graphs. Yield extraction increased with increasing ethanol concentration up to 60%, but decreased sharply with prolonged increase in ethanol concentration, whereas extraction time and temperature had an insignificant effect (Figure 2a–c). It can be observed that with an increase in ethanol concentration from 30 to 60%, there was an increase in EY from 15.0 to 19.5%, when MAE was applied at temperature 70 °C for approximately 15 min.

However, with a further increase from 60 to 100% ethanol, there was a subsequent decrease in EY to 5.6%. These findings agree with those of Yanchev et al. [54], who reported that the extraction yield for *S. scardica* extracts using 70% ethanol under MAE process was approximately 15%. However, lower EY values (10–13%) were reported by Alipieva et al. [33] who studied the MAE parameters that influence the yield of phenolic content from *S. scardica*. According to Figure 2d, TPC decreased with ethanol concentration up to 60%, but beyond this concentration, TPC levels sharply increased from 60 to 100% ethanol, reaching a maximum level of 109.8 mg GAE/g dw. Based on Figure 2e,f, increasing microwave temperature and extraction time resulted in higher yields of TPC. It is noteworthy that the highest TPC was achieved with the highest levels of ethanol concentration, microwave temperature, and extraction time, indicating that these conditions were favorable for the extraction of TPC from *S. scardica* aerial parts.

Similarly, Šavikin et al. [45] indicated from the response surfaces that an increase in ethanol concentration to approximately 80% had an increasing effect on TPC, and a further increase in ethanol concentration led to its slight decrease, when UAE was applied to *S. raeseri* aerial plants. On the contrary, Sarakatsianos et al. [37] found that the best ethanol concentration for the microwave-assisted polyphenols extraction from the *S. raeseri* and *S. scardica* was 60%. The influence of microwave temperature and microwave irradiation time was also reported for other plant species. Hihat et al. [55], reported the significance of microwave power and irradiation time on the yield of TPC from *Coriandrum sativum* leaves.

Moreover, as noticed by the TPC response surfaces, increasing ethanol concentration and microwave temperature resulted in higher yields of ISC DER and HYP DER contents, while extraction time caused an insignificant effect (Figure 2g–l). From Figure 2i, it can be seen that the ISC DER content increased with increasing ethanol concentration to about 100% at the highest level of microwave temperature at 100 °C, reaching a maximum value of 90 mg/g dw when the extraction time was at the central point equal to 15 min. The same trend was also observed in the HYP DER contents of *S. scardica* extracts which reached its maximum value of 113.8 mg/g dw (Figure 2l). These observations have also been reported in the literature during the UAE of polyphenolics from *S. raeseri* extract [45], reporting that temperature extraction had a positive influence on the extraction of both ISC DER and HYP DER. However, they found that the ISC DER and HYP DER contents increased with increasing ethanol concentration to about 80%, and further increase led to a slight decrease.

### 2.4. Optimization of Extraction Parameters and Model Validation

The optimal extraction conditions for obtaining the *S. scardica* extract with maximal content of investigated responses (EY, TPC, ISC DER, and HYP DER contents) were as follows: ethanol concentration of 87.9%, time extraction of 25 min and temperature extraction of 100 °C (Table 4). The optimal conditions of MAE treatment led to predicted EY of 12.29%, TPC of 121.76 mg GAE/100 g dw, ISC DER of 81.87 mg/g dw, and HYP DER of 113.50 mg/g dw. The validation of the predicted values obtained via the models was checked by applying the selected optimal conditions and obtaining experimental results. It was shown that the obtained experimental results were within the 95% confidence interval of predicted values. This confirms that the selected RSM model was successfully applied for the MAE of *S. scardica* in order to obtain extracts with maximal EY, TPC, and contents of ISC DER and HYP DER.

### 2.5. Phytochemical Characterization of Phenolic Extracts from Different Sideritis Species

**Figure 3:**
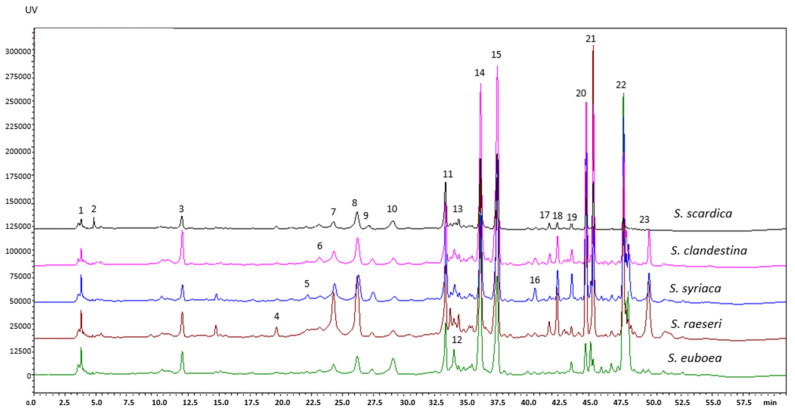
Chromatograms at 280 nm of different *Sideritis* extracts obtained via MAE at optimum conditions. Peak numbers are as those specified in Table 5.

**Figure 4:**
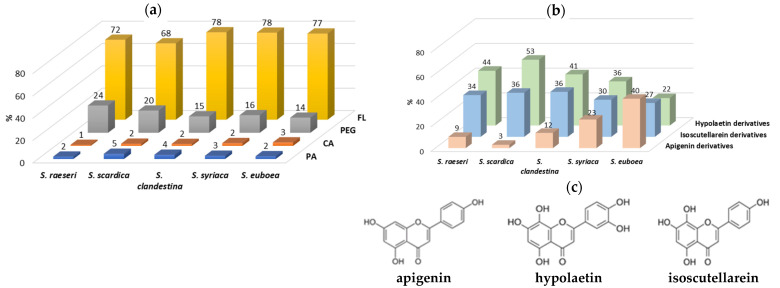
Main categories of phenolic compounds (**a**) and major flavonoids (**b**) identified in the studied *Sideritis* species with their chemical structures (**c**).

In this study, five *Sideritis* species including *S. scardica*, *S. raeseri*, *S. clandestina*, *S. syriaca*, and *S. euboea* were used for the extraction of valuable phenolic compounds using MAE. The phytochemical profile of all *Sideritis* extracts, as determined via HPLC-DAD-ESI-MS analysis in negative ion mode, were similar (Figure 3). Some of the main peaks were tentatively identified and characterized based on their retention times, UV spectra, and MS spectra ([M−H]^−^ ions), as confirmed by comparison with authentic standards or literature data on the *Sideritis* genus (Table 5). In total, of the extracts of the five *Sideritis* species examined herein, twenty-one phytochemicals were successfully identified, including eleven flavonoids (FL), seven phenylethanoid glycosides (PEG), two phenolic acids derivatives (PA) and one carboxylic acid (CA), while two peaks (2 and 19) were unidentified (Figure 4a). In our study, about 72–78% of the total identified phenolic compounds were classified as flavonoids, followed by phenylethanoid glycosides (14–24%), while PA and CA (2–5%) were minor categories. Among all *Sideritis* extracts, those of *S. scardica* and *S. raeseri* contained less FL and more PEG than the other *Sideritis* extracts.

Flavonoids and their derivatives are the dominant phytochemicals, occurring as glycosides, acetyl glycosides, and methylated forms, which are characteristic of the *Sideritis* species [23,24,25,45,51]. The main flavonoid derivatives found in the studied *Sideritis* extracts were HYP, ISC, and apigenin derivatives, and contributed to 22–53%, 27–36%, and 3–40%, respectively, of the total flavonoids identified (Figure 4b). In the *S. raeseri*, *S. scardica*, and *S. clandestina* extracts, HYP derivatives were the main group of components (44, 53, and 41% of the total identified flavonoids, respectively) followed by ISC (34, 36, and 36% of the total identified flavonoids, respectively), and apigenin derivatives (9, 3, and 12% of the total identified flavonoids, respectively). Similar profiles appeared in the *S. syriaca* extract; however, it contained higher apigenin derivatives (23% of the total identified flavonoids) compared to *S. raeseri*, *S. scardica*, and *S. clandestina* extracts. It is worth mentioning that in the *S. euboea* extract, apigenin derivatives were the predominant phenolic compounds (40% of the total identified flavonoids), followed by ISC (27% of the total identified flavonoids), and HYP derivatives (22% of the total identified flavonoids).

Peaks 10 (isoscutellarein-7-O-allosyl(1→2)glucoside), 14 (isoscutellarein-7-O-allosyl-(1→2)-[6″-O-acetyl]-glucoside, 16 (4′-O-methylisoscutellarein-7-O-allosyl(1→2)glucoside), 20 (isoscutellarein-7-O-[6‴-O-acetyl]-allosyl-(1→2)-[6‴-O-acetyl]-glucoside), and 23 (4-O-methyl-isoscutellarein-7-O-[6‴-O-acetyl]-allosyl-(1→2)-[6″-O-acetyl]-glucoside with [M−H]^−^ at *m*/*z* 609, 651, 623, 693, and 707 and similar UV spectra with a maximum absorbance at 230, 276, 306, 328 nm, have been characterized as ISC DER [25,33,35,49,50,51,53,56]. Peaks 11 (hypolaetin-7-O-[6‴-O-acetyl]-allosyl(1→2)glucoside}, 15 (4-O-methylhypolaetin-7-O-[6‴-O-acetyl]-allosyl(1→2)-glucoside), 17 (3-O-methylhypolaetin-7-O-[6‴-O-acetyl]-allosyl(1→2)-glucoside), 18 (hypolaetin-7-O-[6‴-O-acetyl]-allosyl-(1→2)-[6″-O-acetyl]-glucoside) and 21 (3-O-methylhypolaetin-7-O-[6‴-O-acetyl]-allosyl-(1→2)-[6″-O-acetyl]-glucoside) produced deprotonated molecular ions [M–H]^−^ at *m*/*z* 667, 681, 681, 709, and 723, respectively, with similar maximum UV spectra at 252, 277, 302, 338 nm identified as HYP DER [33,35,49,50,51,53,56]. PEG is another group of phenolic derivatives, characteristic of the genus *Sideritis* [49,50,56]. Peaks 6 and 8 had precursor ions at *m*/*z* 623 with UV maximum absorption at 243, 280, 327 nm, and were identified as verbascoside isomer and verbascoside (standard solution), respectively. Peaks 12 and 13 with precursor ions at *m*/*z* 637 as well as UV maximum absorption at 243, 278, 331 nm were identified as leucoseptoside A isomers [49,50,51,52,56]. Peaks 5 and 7 with precursor ions at *m*/*z* 755 as well as UV maximum absorption at 250, 286, 333 nm were characterized as forsythoside B/lavandulifolioside [49,50,51,52,56]. Chlorogenic acid (peak 3) with *m*/*z* 353 (standard) and N1, N10-Bis(p-coumaroyl) spermidine (peak 9) with *m*/*z* 436, were grouped as PA derivatives and identified in all *Sideritis* samples.

### 2.6. Phytochemical Components and Antioxidant Activity of Different Sideritis Species

**Figure 5:**
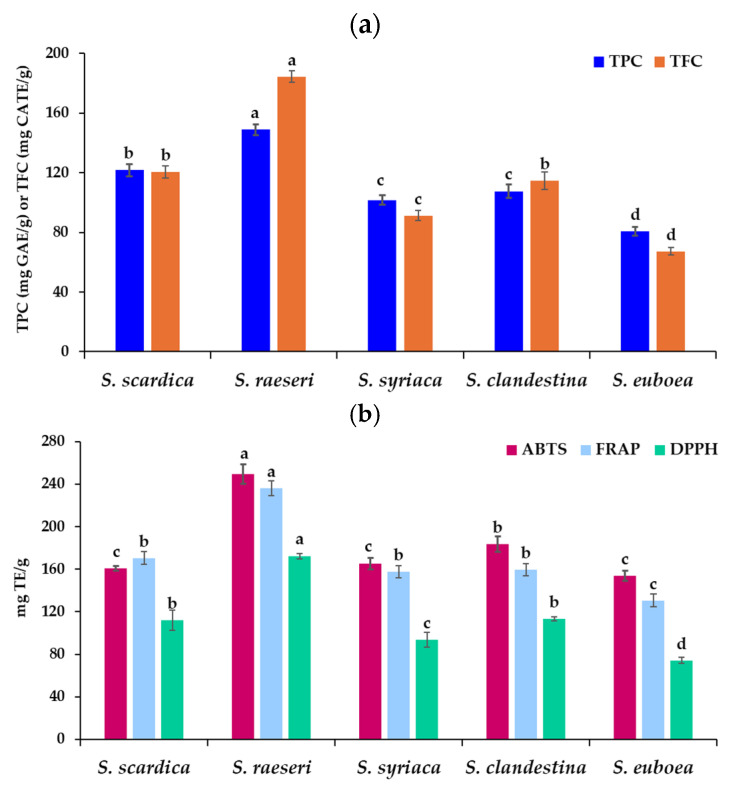
(**a**) Total phenolic content (TPC), total flavonoid content (TFC) and (**b**) antioxidant activity of the obtained extracts from different *Sideritis* species as evaluated via 2,2′-azinobis-(3-ethylbenzothiazoline-6-sulfonic acid (ABTS˙+), 2,2-diphenyl-1-picrylhydrazyl (DPPH˙), and ferric-reducing antioxidant power (FRAP). Different letters in the columns of the same color indicate that there was a significant difference at the corresponding probability level via Tukey′s test (*p* < 0.05).

The bioactive potential of phenolic compounds, a significant class of secondary plant metabolites, is associated with their antioxidant properties. Phenolic extracts from *Sideritis* species are plentiful sources of bioactive chemicals, as has been previously documented. The amount of TPC as well as of total flavonoid content (TFC) of the five *Sideritis* extracts is shown in Figure 5a. It can be noticed that the *S. raeseri* extract had the greatest TPC (149.04 mg GAE/g dw) and TFC (184.52 mg CATE/g dw) values, whereas the *S. euboea* extract had the lowest TPC (80.59 mg GAE/g dw) and TFC (67.31 mg CATE/g dw) values. According to our results, the TPC of five *Sideritis* extracts decreased in accordance with the following order: *S. raeseri* > *S. scardica* > *S. syriaca, S. clandestina > S. euboea*, while the TFC followed a similar order, with minor differences: *S. raeseri* > *S. scardica, S. clandestina* > *S. syriaca > S. euboea*. Previous comparisons among *Sideritis* species also found that *S. raeseri* samples had the highest TPC and TFC followed by *S. scardica* and *S. clandestina* samples [56]. However, the values of TPC and TFC were in accordance with [56] or lower than previous studies [37,57], which may be due to the different extraction method applied and the chemical profile of the original material as well. To the best of our knowledge, few reports are available that employ MAE to extract phenolics from *Sideritis* species. Sarakatsianos et al. [37] reported similar TPC values of 30 mg GAE/g in *S. scardica* and *S. raeseri* samples, obtained via MAE with 60% ethanol as an extraction solvent under 10 min of microwave irradiation time.

Rapid in vitro antioxidant assays, such as direct scavenging of DPPH and ABTS radicals and the capacity of ethanolic extracts to reduce Fe^3+^ ions, were used to assess the antioxidant activity of *Sideritis* extracts. The results of antioxidant activity assays showed the same pattern among the *Sideritis* species, and are presented in Figure 5b. Experimental ABTS values varied from 153.63 to 249.39 mg TE/g dw between *Sideritis* species, while DPPH and FRAP values ranged from 74.20 to 172.44 mg TE/g dw and 130.61 to 236.27 mg TE/g dw, respectively. Since phenolics are the primary antioxidant components in the *Sideritis* ethanolic extracts, the results are in line with those of the TPC. Similarly, *S. raeseri* extract presented the highest antioxidant activity in all three assays, followed by *S. clandestina,* while *S. euboea* presented the lowest antioxidant activity.

Extracts of *S. syriaca* and *S. scardica* had similar and insignificant (*p* > 0.05) ABTS and FRAP values, while the DPPH value of *S. scardica* extract was higher than that of *S. syriaca*. In accordance to our results, regarding FRAP and DPPH values, *S. raeseri* aqueous extracts obtained via the UAE technique and acidic pretreatment showed better antioxidant potential than *S. scardica* according to Dimaki et al. [56]. However, in contrast to our results, the same researchers found that the antioxidant activity of *S. clandestina* was 3- to 4-fold lower than *S. scardica* and *S. raeseri* extracts, belonging possibly to different ecotypes, than our samples. Highly significant correlation was observed between TPC and TFC (r = 0.973, (*p* < 0.001). Similarly, correlation coefficients among TPC and TFC with antioxidant traits were >0.829 which could be considered very highly correlated (Table 6). In accordance with our results, Dimaki et al. [56] reported that FRAP and DPPH values correlated strongly with phenolics and flavonoids in *Sideritis* extracts.

**Figure 6:**
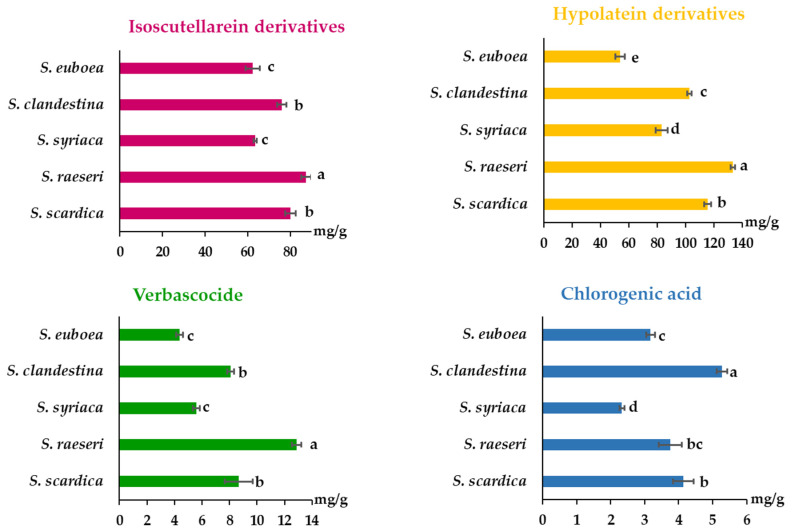
Contents of major phenolic compounds of different *Sideritis* species. Bars capped with the same letter were not significantly different (*p* > 0.05) from each other as determined via Tukey’s test.

The concentration of the key bioactive components is one of the main factors that determines the biological activity, such as antioxidant properties, of the *Sideritis* extracts [58]. However, the present results indicate also that although some of the *Sideritis* extracts have similar quantities of total phenolics, their bioactivity or ability to scavenge free radicals may differ slightly. The existence of the specific phenolic component groups in the respective plant species and their concentration in the extracts provide an explanation for this. After identification via LC-MS and quantification of the target phenolic components, their contents in each *Sideritis* species are shown in Figure 6. Mroz et al. [49] also reported that the major secondary metabolites’ groups in *S. raeseri* and *S. scardica* extracts were classified as flavonoids, terpenoids, phenylethanoid glycosides, and phenolic acids.

According to our results, the major phenolic compounds in all *Sideritis* species, except for *S. euboea* extract, were the HYP DER, followed by ISC DER. Specifically, the contents of ISC DER and HYP DER ranged from 62.51 to 87.55 mg/g dw and 53.78 to 133.21 mg/g dw, respectively. With regard to the HYP DER, the *S. raeseri* extract had the highest content, followed by *S. scardica*, *S. clandestina*, *S. syriaca*, and *S. euboea*. The same trend was observed for the ISC DER contents. Similarly, Dimaki et al. [56] reported that the level of polar metabolites, including HYP DER, was found to be the highest in the *S. raeseri* extract, followed by *S. scardica*, and *S. clandestina*; they have reported that HYP glycosylated derivatives had the strongest presence, especially in *S. raeseri* and *S. scardica* samples (108.02 and 96.73 mg/100 g of dry plant material, respectively). Contrasting with our results, Zheleva-Dimitrova et al. [59] reported that ISC derivatives were more abundant than HYP derivatives in lyophilized *S. scardica* water extracts (infusions). Similarly, Sarrou et al. [60] reported that total ISC DER were present in higher amounts than HYP DER, ranging from 1372.6 to 2094.8 mg/100 g and 954.0 to 1247.6 mg/100 g, respectively, in three *S. scardica* clones. According to Mroz et al. [49], the concentration of ethanol in the solvent (hydroethanolic mixtures) affects the extraction efficiency of major classes of bioactive compounds in *S. scardica* and *S. raeseri*, and additionally, the difference in phenolic profile between them is related to their terpenoid profile. In addition, several factors that can affect the biosynthesis of specialized products including harvesting time, environmental conditions, the type and origin of the plant material (e.g., fresh or dried/cultivated or collected from wild populations, plant parts), and their tendency to spontaneously hybridize are known to cause a significant degree of heterogeneity in *Sideritis* essential oils′ chemical composition, as it has been demonstrated via phytochemical research [61].

Verbascoside (VER) was found to be the third major compound in *Sideritis* extracts ranging from 4.37 to 12.86 mg/g dw, with *S. raeseri* appearing as the highest content among the five species, followed by *S. scardica*, *S. clandestina*, *S. syriaca*, and *S. euboea*. In their study, Dimaki [56] reported VER values of 52.53 ± 5.22 mg rutin equivalents/100 g dry plant material for *S. raeseri* aqueous extracts produced via the UAE process using acidic pretreatment, while *S. scardica* extracts contained 39.05 ± 2.31 mg rutin equivalents/100 g dry plant material and *S. clandestina* in trace. Sarrou [60] reported that hydromethanolic extracts (80% MeOH), produced by shaking, of *S. scardica* clones had VER values ranging from 1511.51 to 2234.32 mg/100 g. Chlorogenic acid (CL) was the fourth major compound in our samples, its content ranging from 2.33 to 5.27 mg/g dw, with *S. clandestina* having the significantly highest content (*p* < 0.05), followed by *S. scardica*, *S. raeseri*, *S. euboea*, and *S. syriaca*. Chlorogenic acid, along with other phenolic acids, were reported to be present in *S. scardica* infusions [16], whereas chlorogenic acid and numerous ISC derivatives were found previously in extracts of *S. syriaca* [27]. Kaparakou et al. [50] found in *S. raeseri* hydromethanolic extracts obtained via UAE, that flavonoids (i.e., ISC, HYP, and apigenin) were the main group components, followed by phenylethanoid glycosides (i.e., verbascoside) and phenolic acid (i.e., chlorogenic acid). All of these species are closely related and may provide similar health benefits, as evidenced by their similar polyphenolic patterns.

**Figure 7:**
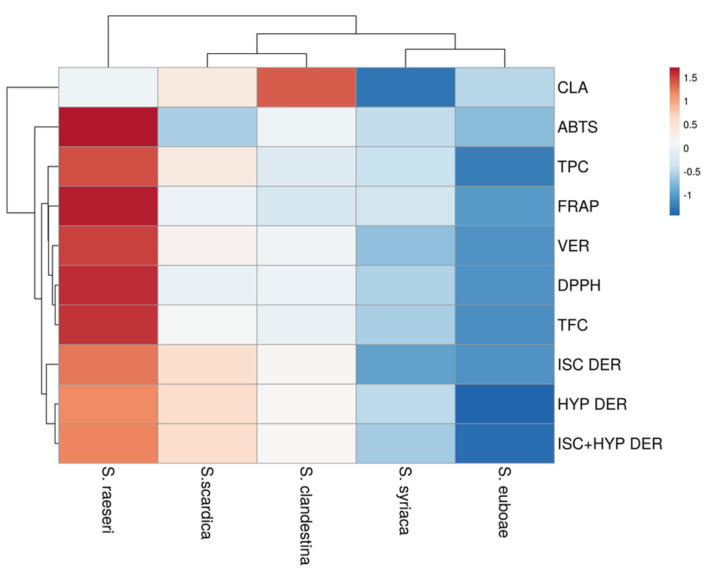
Heatmap representing the varying content of *Sideritis* species in chlorogenic acid (CLA), verbascocide (VER), hypolaetin (HYP) and isoscutellarein (ISC) derivatives, antioxidant activity as estimated via DPPH, ABTS, and FRAP assays, total phenolic content (TPC), and total flavonoid content (TFC).

Furthermore, *Sideritis* extracts were grouped into two main clusters based on their antioxidant activity, TPC, and TFC contents, and contents in major phenolic compounds (CLA, VER, HYP, and ISC derivatives) via hierarchical clustering, which is visualized in the heatmap (Figure 7). The first cluster consisted solely of *S. raeseri* extract, which showed the highest content in TPC, TFC, DPPH, ABTS, and FRAP values, as well as all major phenolics except CLA. The extracts of the remaining *Sideritis* species were divided into two subgroups in the second group. More precisely, the *S. scardica* and *S. clandestina* extracts were in the first subgroup. They had medium levels of ISC and HYP DER, VER, TPC, and TFC, as well as medium to low levels of antioxidant activity. Their CLA content was also rather high, with SC having the highest level of any extract. SY and SE extracts were located in the second subgroup; of all the species evaluated, they had the lowest levels of TPC, TFC, antioxidant activity, and phenolic content.

## 3. Materials and Methods

### 3.1. Materials and Chemicals

Five *Sideritis (S)* species growing in Greece were investigated in the present study: *S. scardica S. raeseri,* and *S. euboea*, originated from cultivated accessions, in the Hellenic Agricultural Organization—Dimitra, Institute of Plant Breeding and Genetic Resources (Thermi, Thessaloniki, Greece), *S. syriaca* was obtained from cultivated plants in Creta, and *S. clandestina* subsp. *peloponnesiaca*, was collected from Mt. Menalon, (Peloponnese region, south Greece). All the samples including the aerial flowering parts, were collected in 2023. The fresh plant material, consisting of stalks, leaves, and flowers, were dried at room temperature and stored under controlled environmental conditions (25 °C) until extraction. Dried plant material was ground in a laboratory mill (Fritsch Milling & Sizing, Inc., Model Pulverisette 11, Pittsboro, NC, USA) prior to extraction.

Analytical standard verbascoside (VER) was obtained from Sigma-Aldrich (Steinheim, Germany); quinic acid (QA), and chlorogenic acid (CLA), were purchased from Extrasynthese (Genay Cedex, France). The analytical reagents 2,2-diphenyl-1-picryhydrazyl (DPPH), 2,2-azinobis-(3-ethylbenzthiazoline-6-sulphonic acid) (ABTS), and 2,4,6-tripyridyl-s-triazine (TPTZ) were purchased from Sigma-Aldrich (Steinheim, Germany), as well as formic acid, methanol, acetonitrile, and water of LC-MS grade. Ethanol (96%), which is regarded as a GRAS (generally recognized as safe) solvent for use in the food industry, was employed for the extraction of antioxidants. All the other solvents used for the extraction of phenolic compounds as well as the chromatographic analysis were of HPLC or LC-MS grade.

### 3.2. Isolation of Isoscutellarein 7-O-Allosyl-(1→2)-(6‴-O-Acetyl)-Glucoside

For the isolation of isoscutellarein 7-O-allosyl-(1→2)-(6‴-O-acetyl)-glucoside (ISC) the closely related Stachys recta was used, as previously reported [62]. In brief, lyophilized ethanol extract from the leaves of *S. recta* (15.5 g) was redissolved in 200 mL of water/methanol (4:1, *v*/*v*), defatted with cyclohexane (organic phase A), and then partitioned (in triplicate) with equal volumes of cyclohexane/diethylether (1:3, *v*/*v*) (organic phase B), and with a mixture of ethyl acetate/cyclohexane (4:1, *v*/*v*) (organic phase C). The remaining polar phase was partitioned with butanol to yield 6.5 g of butanol extract (organic phase D) and the aqueous phase after lyophilization afforded approximately 5.0 g of extract E (phase E). A small part of the organic phase D (~ 2 g) was subjected to column chromatography over Sephadex LH-20 with MeOH 50% and afforded six subfractions (DA-DF). Subfraction DC (480.2 mg) was fractionated successively over Sephadex LH-20 with MeOH 50% and afforded 37.5 mg of ISC of purity 78% (normalization HPLC).

### 3.3. Microwave-Assisted Extraction (MAE)

MAE was carried out in an advanced microwave extraction system Millestone ETHOS X (Milestone, Sorisole, Italy). It consisted of a multimode microwave reactor equipped with two magnetrons (950 W of each) for microwave irradiation and an infrared sensor monitoring the temperature. The corresponding panel managed the temperature and extraction duration. The experiments were carried out at atmospheric pressure using capped TFM jars (maximum volume of 100 mL) that were positioned separately in the appropriate rotor locations. Prior to extraction, portions of 0.25 g of powdered *Sideritis* materials were put in TFM jars, mixed with 20 mL of aqueous ethanol solutions at different concentrations. An infrared easyTEMP sensor was positioned on the microwave cavity’s bottom to measure the temperature within the vessels. After extraction, under the guidelines outlined in the experimental design (Table 3), the extracts were cooled for 10 min. Once the extraction process was finished, the extracts were filtered through Whatman No. 1 filter paper lining a Büchner funnel and the filtrates were collected in a volumetric flask. Then, the ethanolic extracts was evaporated in a rotary evaporator (Heidolph Instruments GmbH & Co. KG, Schwabach, Germany) at 40–60 °C under vacuum, until the removal of ethanol. The remaining aqueous extract and the washings were subsequently freeze-dried (Christ, Martin Christ Gefriertrocknungsanlagen GmbH, Osterode am Harz, Germany) for 48 h. The dried extracts were weighed and stored at −25 °C until further analyses. The extraction yield (EY) of *Sideritis* samples was calculated according to the following formula:

(5) $$EY\;\left(\%\right)=\frac{weight\;of\;freeze-dried\;extract\;\left(g\right)}{weight\;of\;pretreated\;Sideritis\;powder\;\left(g\right)}\times 100$$

### 3.4. LC-DAD-MS Analysis

Extracts of different *Sideritis* species were analyzed via the Nexera HPLC system (Shimatzu, Kyoto, Japan) equipped with a Poroshell 120 EC-C_18_ column (4.6 × 150 mm, 4 µm), diode array detector, and a single quadrupole mass spectrometer (LCMS-2020) (Shimatzu, Kyoto, Japan). The system was equipped with an ESI ion source and the mass range was *m*/*z* 100–1000 in full scan mode. Working conditions were in ESI negative mode. The chromatographic system was controlled with Lab Solutions 5.97.SP1 software (Shimadzu, Kyoto, Japan). The mobile phase consisted of solvent A (0.1% *v*/*v* solution of formic acid in water) and solvent B (acetonitrile). Separation was achieved according to the following gradient scheme: 90–85% A, 0–7 min; 85–82% A, 7–12 min; 82% A, 12–25 min; 82–75% A, 25–27 min; 75%A 27–32 min; 75–60% A, 32–42 min; 60%A, 42–49 min; 60–90% A, 49–53 min; and 90% A, 53–60 min. The flow rate of 0.4 mL/min was used in all separations, while the column temperature was maintained at 30 °C. The injection volume was 10 μL. Interface temperature and DL temperature were 350 °C and 250 °C, respectively. Nitrogen was used as the gas for ionization. The identification of the phenolic compounds was based on comparison of their retention time and their obtained mass and UV spectra to the literature data. Quantification was carried at 280 and 330 nm, constructing calibrations curves of corresponding standard solutions of chlorogenic acid, verbascoside, and ISC at five concentration levels within the linear range of 0.1 to 100 μg/mL. Correlation coefficients (r^2^) from calibration curves for all the compounds were between 0.9981 and 0.9990. The limit of detection was in the range of 0.076 to 0.093 μg/mL and the limit of quantification was from 0.232 to 0.264 μg/mL. The results of intra-day and inter-day precision were less than 3.1% and 8.2%, respectively. Τhe contents of HYP derivatives are expressed as ISC equivalents. Analyses were performed in triplicate and the results are expressed as milligrams per gram of lyophilized extract (mg/g dw).

### 3.5. Total Phenolic Content (TPC), Total Flavonoids Contents (TFC), and Antioxidant Activity

TPC was determined with the Folin–Ciocalteau reagent method [63] at 725 nm and is expressed as mg of gallic acid equivalents (GAE) per g of lyophilized extract (mg GAE/g dw). TFC was determined via the aluminum chloride (AlCl_3_) method [64] at 510 nm and the results are expressed as mg of catechin equivalents (CATE) per g of lyophilized extract (mg CATE/g dw). The antioxidant activity was evaluated with three different assays: 2,2-diphenyl-1-picrylhydrazyl (DPPH) radical scavenging activity, 2,2-azinobis-(3-ethylbenzthiazoline-6-sulphonic acid) (ABTS) radical scavenging activity, and ferric reducing antioxidant power (FRAP). Briefly, in the DPPH method [65], which measures the ability of antioxidants to scavenge the stable radical DPPH, the absorbance of extract was measured at 516 nm; the inhibition of DPPH (%) was calculated by using the following equation:

(6) $$Inhibition=\left[\frac{A0-As}{A0}\right]\times 100$$

and the results are reported as mg Trolox equivalent per g of lyophilized extract (mg TE/g dw). In the ABTS method [66], the absorption was recorded at 734 nm, the inhibition of the ABTS radical cation (%) was calculated as described in Equation (6) and the results are reported as mg Trolox equivalent per g of lyophilized extract (mg TE/g dw). According to FRAP method [67] which measures the ability of antioxidants to reduce the [Fe^III^(TPTZ)_2_]^3+^ to [Fe^II^(TPTZ)_2_]^2+^, the absorption was recorded at 593 nm and the results are expressed as mg Trolox equivalent per g of lyophilized extract (mg TE/g dw).

### 3.6. Experimental Design and Optimization

A Box–Behnken experimental design (BBD) composed of 15 runs with three replicates was selected to determine optimal ΜAΕ conditions. Ethanol concentration (X_1_, %), extraction time (X_2_, min), and extraction temperature (X_3_, °C) were independent variables that were used to investigate the effect of extracts on EY, TPC, ISC DER, and HYP DER contents. Table 1 summarizes the factors and levels investigated in the experimental design. The factors were normalized through the coding of the independent variables and their range from +1 to −1 to affect the response more evenly. Experiments were randomized in order to minimize the systematic bias in the observed responses due to extraneous factors. A full quadratic mathematical model was fitted to each response variables, according to the following equation:

(7) $$Y=\beta_{0}+\sum_{i=1}^{k}\beta_{i}X_{i}+\sum_{i=1}^{k}\sum_{j=2}^{k-1}\beta_{ij}X_{i}X_{j}+\sum_{i=1}^{k}\beta_{ii}X_{i}^{2}$$

where Y is the measured response, X_i_ and X_j_ are the independent variables affecting the response and β_0_, β_i_, β_ii_, and β_ij_ are the regression coefficients for intercept, linear, quadratic and interaction terms, respectively.

Model analysis was carried out by employing analysis of variance (ANOVA) with significance levels set at 0.05. The adequacy of the model(s) was calculated using the coefficient of determination (R^2^), the adjusted determination coefficient (R^2^_adj_), the adequate precision, *p*-value of the models, and the lack of fit. The desirability function was used to optimize extraction conditions (X_1_, X_2_, and X_3_) calculating the maximum values of EY, TPC, ISC DER, and HYP DER as responses. Optimal MAE conditions were used to fit the experimental data.

### 3.7. Statistical Analysis

Design matrix, fitting, statistical analysis, and optimization of the MAE process were calculated via Design-Expert Software, Version 13 (Stat Ease, Inc., Minneapolis, MN, USA). One-way analysis of variance (ANOVA) combined with Tukey′s test was used to test for differences among different *Sideritis* species, using Minitab version 18 (Minitab, Inc., State College, PA, USA). For all statistical tests, differences at *p* < 0.05 were considered significant.

## 4. Conclusions

In the current study, the polyphenolic compounds from five Greek *Sideritis* species (*S. scardica*, *S. clandestina*, *S. raeseri*, *S. euboea*, and *S. syriaca*) using the Microwave-Assisted Extraction (MAE) process were investigated. For the optimization of the MAE parameters, we evaluated the effect of ethanol concentration, time extraction, and microwave temperature to improve the EY, TPC, and contents of target phenolic components extracted via MAE from *S. scardica* using the response surface methodology. We present an environmentally friendly approach, for the first time, with the emphasis on the maximization of ISC and HYP derivatives that are characteristic phenolic compounds of the *Sideritis* species. Ethanol concentration was the most significant factor affecting the EY and phenolic components in MAE, while temperature and extraction time were less important. The optimal MAE conditions for maximization of the responses were as follows: ethanol concentration 87.9%, microwave irradiation time 25 min, and microwave temperature 100 °C. Under these optimal parameters, the developed model predicted high levels of the investigated responses that were confirmed by the experimental results. Then, the optimal MAE conditions were applied to five *Sideritis* taxa that are native to Greece (*S. clandestina*, *S. raeseri*, *S. euboea*, *S. syriaca*, and *S. scardica*) to compare their phytochemical components that were highly correlated to antioxidant potential as evaluated via DPPH, ABTS, and FRAP assays. The results revealed that all *Sideritis* extracts showed a similar phytochemical profile, however, significant differences in the contents of the main phenolic compounds were noticed among them. In particular, hierarchical clustering analysis showed a correlation between the bioactive compounds studied and the *Sideritis* species. Our findings indicate that *Sideritis* extracts have the potential to be used as natural antioxidants in food and nutraceutical products with pronounced antioxidant properties. In addition, MAE could be an effective and environmentally friendly procedure since it accelerates the extraction of valuable phenolics from the *Sideritis* species.

## Figures and Tables

**Table 1:** Coded and actual levels of independent factors employed in RSM for optimization of Microwave-Assisted Extraction (MAE).

Factors	Symbol	Levels		
		−1	0	+1
Ethanol concentration (% (*v/v*))	*X*_1_	30	65	100
Extraction time (min)	*X*_2_	5	15	25
Extraction temperature (°C)	*X*_3_	30	45	60

**Table 2:** Box–Behnken design with MAE parameters and experimental data of extraction yield (EY), total phenolic content (TPC), as well as the contents of isoscutellarein (ISC DER) and hypolaetin (HYP DER) derivatives.

Run	Independent Variables			Responses			
	Ethanol Conc. (%)	Extraction Time (min)	Temperature (°C)	EY (%)	TPC (mg GAE/g dw)	ISC DER (mg/g dw)	HYP DER (mg/g dw)
1	30	15	100	17.5	96.36	54.02	82.03
2	65	15	70	19.6	93.45	62.77	80.60
3	65	15	70	19.6	92.00	60.64	74.49
4	65	25	40	15.3	90.00	76.03	92.04
5	65	15	70	21.6	95.01	60.21	72.03
6	100	15	40	6.3	95.90	75.62	84.14
7	100	15	100	10.4	123.70	92.03	135.61
8	30	15	40	15.2	96.10	68.02	85.86
9	65	5	40	15.0	98.03	71.85	89.04
10	30	25	70	15.5	107.64	69.53	82.01
11	65	25	100	15.8	109.45	70.89	98.83
12	65	5	100	15.8	92.03	75.03	104.32
13	30	5	70	15.2	106.24	65.00	90.02
14	100	5	70	5.6	109.80	80.50	100.70
15	100	25	70	8.6	125.01	90.03	113.78

**Table 3:** ANOVA of MAE parameter optimization of designed quadratic models.

Model Term	DF	EY			TPC			ISC DER			HYP DER		
		SS	F-Value	*p*- Value	SS	F-Value	*p*- Value	SS	F-Value	*p*-Value	SS	F-Value	*p*- Value
model	9	306.91	23.79	0.0014 **	1693.19	23.29	0.0014 **	1541.94	20.04	0.0021 **	3701.64	15.50	0.0012 **
*X*_1_	1	131.69	91.88	0.0002 ***	283.62	35.27	0.0019 **	832.12	97.35	0.0002 ***	1148.85	43.30	0.0012 **
*X*_2_	1	1.83	1.27	0.3101	84.77	10.54	0.0228 *	24.64	2.88	0.1503	0.1495	0.0056	0.9431
*X*_3_	1	7.44	5.19	0.0717	210.94	20.13	0.0037 ***	0.0253	0.0030	0.9587	608.27	22.92	0.0049 *
*X*_1_^2^	1	135.21	94.34	0.0002 ***	542.07	67.41	0.0004 ***	183.08	21.42	0.0057 **	413.40	15.58	0.0109 *
*X*_2_^2^	1	32.22	22.48	0.0051 **	159.07	19.81	0.0067 **	241.01	28.20	0.0032 **	348.56	13.14	0.0152 *
*X*_3_^2^	1	12.21	8.52	0.0330 *	26.74	3.33	0.1237	65.96	7.72	0.0390 *	416.65	15.70	0.0107 *
*X*_1_ *X*_2_	1	1.79	1.25	0.3140	47.60	26.23	0.0592	6.18	0.7224	0.4341	139.07	5.24	0.0707
*X*_1_ *X*_3_	1	0.7657	0.5343	0.4976	183.78	5.92	0.0050 *	230.74	26.99	0.0035 **	765.39	28.84	0.0030 **
*X*_2_ *X*_3_	1	0.0268	0.0187	0.8966	161.89	22.85	0.0065 **	17.18	2.01	0.2155	18.03	0.6794	0.4473
lack of Fit	3	4.46	1.10	0.5092	35.71	5.29	0.1631	38.53	6.11	0.1439	93.47	1.59	0.4087
pure error	2	2.71			4.5			4.21			39.21		
R^2^		0.9772			0.9768			0.9730			0.9654		
R^2^_adj_		0.9361			0.9351			0.9245			0.9031		
adeq. pr.		14.5373			15.4223			14.9079			13.2526		

MAE, Microwave-Assisted Extraction; EY, extraction yield; TPC, total phenolic content; ISC DER, isoscutellarein derivatives; HYP DER, hypolaetin derivatives; *X*_1_, *X*_2_ and *X*_3_ are values of independent variables: ethanol concentration (%), extraction time (min), and extraction temperature (°C) respectively.; R^2^_adj_, adjusted R^2^; adeq. pr, adequate precision; *, **, *** indicates a significant term at *p* < 0.05, *p* < 0.01 and *p* < 0.001, respectively.

**Table 4:** Predicted and actual response values for numerical optimization of MAE conditions.

Optimized Conditions	Response	Predicted Values	95% CI Low for Mean	95% CI High for Mean	Experimental Values	Desirability
Ethanol: 87.9% Time: 25 min Temperature: 100 °C	Yield (%)	12.29 ± 1.20	9.27	15.30	14.07 ± 0.38	0.762
	TPC (mg GAE/g)	127.93 ± 2.84	120.80	135.10	121.76 ± 4.19	
	ISC DER (mg/g)	88.68 ± 2.92	81.33	96.07	81.87 ± 2.89	
	HYP DER (mg/g)	128.10 ± 5.15	115.15	141.12	118.50 ± 6.36	

Experimental data are expressed as mean ± standard deviation (*n* = 3); CI, confidence interval.

**Table 5:** Phytochemical composition of *Sideritis* extracts; SR (*S. raeseri*), SS (*S. scardica*), SC (*S. clandestina*), SY (*S. syriaca*) and SE (*S. euboea*).

No.	Compound	RT (min)	UV λmax (nm)	[M-H]^−^ (*m*/*z*)	% Peak Area					Reference
					SR	SS	SC	SY	SE	
1	quinic acid	3.84	-	191	0.8	1.7	1.6	2.3	2.7	standard
2	unknown	4.84	245, 281, 332	637	0.1	1.2	0.7	0.5	0.1	-
3	chlorogenic acid	11.89	291sh, 328	353	1.5	2.9	2.5	1.3	1.2	standard
4	coumaroylmelittoside derivative	19.62	311	711	0.6	0.6	0.5	0.5	0.6	[49]
5	forsythoside/lavandulifolioside	22.11	250, 286, 333	755	1.3	0.6	0.7	1.2	0.7	[49,50,51,52]
6	verbascoside isomer	23.01	291sh, 329	623	0.6	2.4	1.6	1.4	1.1	[49,50,52]
7	forsythoside/lavandulifolioside	24.12	249, 291, 329	755	7.4	2.8	3.0	3.8	2.5	[49,50,52]
8	verbascoside	26.13	291sh, 329	623	8.7	6.8	3.8	3.9	2.9	standard
9	N1, N10-Bis(p-coumaroyl) spermidine	27.40	247, 307	436	0.4	1.1	0.9	1.3	0.9	[49]
10	isoscutellarein 7-O-allosyl(1→2)glucoside	29.02	230, 276, 305, 325	609	0.8	4.5	1.3	0.9	3.2	[44,49,51]
11	hypolaetin 7-O-[6‴-O-acetyl]-allosyl(1→2)glucoside	33.31	253, 276, 296, 339	667	4.7	10.8	4.5	6.1	4.3	[39,50,52]
12	leucoseptoside A isomer	34.10	243, 278, 331	637	1.5	0.9	2.9	3.0	3.8	[49,50,52]
13	leucoseptoside A isomer	34.50	243, 276, 330	637	1.1	1.9	-	-	-	[49,50,52]
14	isoscutellarein 7-O-allosyl-(1→2)-[6″-O-acetyl]-glucoside	36.32	230, 276, 306, 328	651	10.3	14.9	14.6	8.3	14.9	standard
15	4′-O-methylhypolaetin 7-O-[6‴-O-acetyl]-allosyl(1→2)-glucoside	37.91	252, 278, 298, 340	681	11.3	16.9	17.4	13.5	10.3	[49,50,51,52]
16	4′-O-methylisoscutellarein-7-O-allosyl(1→2)glucoside	40.82	230, 278, 302, 325	623	0.4	0.5	0.7	1.2	0.3	[25,49,50,51,52]
17	3′-O-methylhypolaetin 7-O-[6‴-O-acetyl]-allosyl(1→2)-glucoside	41.90	252, 277, 297, 337	681	1.0	1.3	0.9	0.8	0.3	[35,49,50,51]
18	hypolaetin 7-O-[6‴-O-acetyl]-allosyl-(1→2)-[6″-O-acetyl]-glucoside	42.52	254, 278, 297, 338	709	2.8	1.2	1.7	2.2	0.2	[35,49,50,51,53]
19	unknown	43.73	246, 295, 310	598	0.5	1.2	1.4	2.8	0.9	-
20	isoscutellarein 7-O-[6‴-O-acetyl]-allosyl-(1→2)-[6″-O-acetyl]-glucoside	44.90	230, 276, 306, 325	693	7.8	4.4	9.4	10.3	2.2	[25,35,49,51,53]
21	3-O-methylhypolaetin 7-O-[6‴-O-acetyl]-allosyl-(1→2)-[6″-O-acetyl]-glucoside	45.42	256, 276, 298, 339	723	11.8	6.2	7.8	5.2	2.0	[35,49,50,51,53]
22	apigenin-7-O-(6″-O-4-coumaroyl)-glucoside	48.02	232, 276, 317	577	6.5	1.8	9.6	18.0	30.8	[35,49,50,51,54]
23	4-O-methylisoscutellarein 7-O-[6‴-O-acetyl]-allosyl-(1→2)-[6″-O-acetyl]- glucoside	49.81	244, 273, 307, 329sh	707	4.9	0.1	2.4	2.6	0.5	[35,49,50,51]

**Table 6:** Pearson′s correlation matrix among traits.

	TPC	TFC	DPPH	ABTS	FRAP	ISC DER	HYP DER
TPC	1						
TFC	0.973 ***	1					
DPPH	0.945 ***	0.985 ***	1				
ABTS	0.829 ***	0.904 ***	0.932 ***	1			
FRAP	0.954 ***	0.963 ***	0.967 ***	0.925 ***	1		
ISC DER	0.895 ***	0.913 ***	0.897 ***	0.728 **	0.831 ***	1	
HYP DER	0.947 ***	0.927 ***	0.900 ***	0.724 **	0.862 ***	0.935 ***	1

*** *p* < 0.001; ** *p* < 0.01.

## Data Availability

Data are contained within the article.
